# Supplementary material for: Large language models are poor clinical administrators: An evaluation of structured queries in real-world electronic health records
Source: PLOS Digit Health. 2026 May 7;5(5):e0001326. doi: 10.1371/journal.pdig.0001326 (PMC13152155; doi:10.1371/journal.pdig.0001326)
Supplement: S1 List — (DOCX) [file pdig.0001326.s002.docx]

**S1 List:** EdEncounterFact Table Columns Names

'EdVisitKey', 'PatientKey', 'PatientDurableKey', 'AgeKey', 'HospitalAdmissionKey', 'EncounterKey', 'ArrivalDateKey', 'ArrivalTimeOfDayKey', 'AvsPrintDateKey', 'PreviousEdVisitKey', 'NextEdVisitKey', 'FirstAttendingAssignedKey', 'FirstAttendingAssignedDurableKey', 'LastAttendingAssignedKey', 'LastAttendingAssignedDurableKey', 'LongestAttendingAssignedKey', 'LongestAttendingAssignedDurableKey', 'AvsPrintTimeOfDayKey', 'BedRequestDateKey', 'BedRequestTimeOfDayKey', 'BedAssignedDateKey', 'BedAssignedTimeOfDayKey', 'DepartureDateKey', 'DepartureTimeOfDayKey', 'DepartureUTCDateKey', 'DepartureUTCTimeOfDayKey', 'DispositionDateKey', 'DispositionTimeOfDayKey', 'FirstArrivalOutsideEdDateKey', 'FirstArrivalOutsideEdTimeOfDayKey', 'FirstAttendingAssignedDateKey', 'FirstAttendingAssignedTimeOfDayKey', 'AllergiesReviewedDateKey', 'AllergiesReviewedTimeOfDayKey', 'MedicationListReviewedDateKey', 'MedicationListReviewedTimeOfDayKey', 'NonEdPatientClassDateKey', 'NonEdPatientClassTimeOfDayKey', 'RoomedDateKey', 'RoomedTimeOfDayKey', 'EdDepartmentKey', 'PrimaryCareAreaKey', 'FirstNonEdDepartmentKey', 'AdmissionDepartmentKey', 'PrimaryEdDiagnosisKey', 'PrimaryChiefComplaintKey', 'ChiefComplaintComboKey', 'EdDiagnosisComboKey', 'LevelOfServiceAuthorizingProviderKey', 'LevelOfServiceAuthorizingProviderDurableKey', 'AllergiesReviewedUserKey', 'AllergiesReviewedUserDurableKey', 'MedicationListReviewedUserKey', 'MedicationListReviewedUserDurableKey', 'GuarantorKey', 'GuarantorDurableKey', 'CoverageKey', 'ArrivalMethod', 'EdDisposition', 'DischargeDisposition', 'AcuityLevel', 'LevelOfCare', 'FinancialClass', 'FacilityLevelOfService', 'ProfessionalLevelOfService', 'EncounterEpicCsn', 'HospitalAccountEpicId', 'PrimaryProfessionalAccountEpicId', 'ArrivalInstant', 'AvsPrintInstant', 'BedRequestInstant', 'BedAssignedInstant', 'DepartureInstant', 'DepartureUTCInstant', 'DispositionInstant', 'FirstArrivalOutsideEdInstant', 'FirstAttendingAssignedInstant', 'NonEdPatientClassInstant', 'RoomedInstant', 'AllergiesReviewedInstant', 'MedicationListReviewedInstant', 'FacilityCriticalCareLevelOfService', 'FacilityCriticalCareLevelOfService_YesNo', 'ProfessionalCriticalCareLevelOfService', 'ProfessionalCriticalCareLevelOfService_YesNo', 'PresentedWithOpioidOverdose', 'EdVisitPrimaryCareAreaKey', 'EdGenericDispo', 'AdmissionProviderKey', 'AdmissionProviderDurableKey', 'DispositionProviderDurableKey', '_CreationInstant', '_LastUpdatedInstant'
