# Supplementary material for: Large language models are poor clinical administrators: An evaluation of structured queries in real-world electronic health records
Source: PLOS Digit Health. 2026 May 7;5(5):e0001326. doi: 10.1371/journal.pdig.0001326 (PMC13152155; doi:10.1371/journal.pdig.0001326)
Supplement: S1 Table — (DOCX) [file pdig.0001326.s003.docx]

| **AcuityLevel (ESI)** | **N (%)** |
| --- | --- |
| Non-Urgent (5) | 5486 (1.3%) |
| Less Urgent (4) | 71224 (17.5%) |
| Urgent (3) | 239475 (58.8%) |
| Emergent (2) | 80164 (19.7%) |
| Immediate (1) | 2862 (0.7%) |
| *Unspecified | 7869 (1.9%) |

S1 Table: Emergency Severity Index (ESI) Acuity Level Distribution

ESI – Emergency Severity Index
