# Supplementary material for: Large language models are poor clinical administrators: An evaluation of structured queries in real-world electronic health records
Source: PLOS Digit Health. 2026 May 7;5(5):e0001326. doi: 10.1371/journal.pdig.0001326 (PMC13152155; doi:10.1371/journal.pdig.0001326)
Supplement: S2 Table — (DOCX) [file pdig.0001326.s004.docx]

| **ArrivalMethod** | **N (%)** |
| --- | --- |
| By Personal Means | 269634 (66.2%) |
| 911 EMS | 64919 (15.9%) |
| Ambulance (non-911/Private) | 58673 (14.4%) |
| Other | 12047 (3.0%) |
| Police | 1316 (0.3%) |
| Transfer | 358 (0.1%) |
| Correctional Service | 108 (0.0%) |
| Unspecified | 23 (0.0%) |
| Ambulette | 2 (0.0%) |

**S2 Table: Patient Arrival Method Distribution**

Distribution of emergency department arrivals by method of transport, including personal means, emergency medical services (EMS), and other specified categories.
