# Supplementary material for: Large language models are poor clinical administrators: An evaluation of structured queries in real-world electronic health records
Source: PLOS Digit Health. 2026 May 7;5(5):e0001326. doi: 10.1371/journal.pdig.0001326 (PMC13152155; doi:10.1371/journal.pdig.0001326)
Supplement: S3 Table — (DOCX) [file pdig.0001326.s005.docx]

| **EdDisposition** | **N (%)** |
| --- | --- |
| Discharge | 306206 (75.2%) |
| Admit | 65341 (16.1%) |
| Left Without Being Seen | 7775 (1.9%) |
| Transfer | 7707 (1.9%) |
| AMA | 6337 (1.6%) |
| Left Before Treatment Complete | 5966 (1.5%) |
| Unspecified | 1985 (0.5%) |
| Send to Another Department | 1351 (0.3%) |
| Send to L&D | 1210 (0.3%) |
| Premature Departure | 606 (0.1%) |
| Chart Created in Error | 564 (0.1%) |
| Expired | 493 (0.1%) |
| Sent to Clinic | 309 (0.1%) |
| Eloped | 287 (0.1%) |
| Discharge to ER | 225 (0.1%) |
| EOB | 221 (0.1%) |
| Send To | 189 (0.0%) |
| Observation | 128 (0.0%) |
| Discharge from Observation | 91 (0.0%) |
| OR | 20 (0.0%) |
| Admit to EOB | 20 (0.0%) |
| RETU / Observation | 11 (0.0%) |
| Authorized Walkout | 8 (0.0%) |
| CPEP Hold | 7 (0.0%) |
| Transfer to Cath Lab | 5 (0.0%) |
| Send to Medical ER | 5 (0.0%) |
| Transfer to Mount Sinai Hospital Manhattan | 4 (0.0%) |
| Pediatric Observation | 2 (0.0%) |
| Downtime Chart | 2 (0.0%) |
| Admit to Detox | 2 (0.0%) |
| Discharge from Inpatient | 1 (0.0%) |
| Transfer to Mount Sinai West | 1 (0.0%) |
| Send to MSQ ED | 1 (0.0%) |

**S3 Table: Emergency Department Disposition Distribution**

Final dispositions recorded at the conclusion of emergency department visits, including discharges, admissions, transfers, and various forms of patient departure.
