# Supplementary material for: Large language models are poor clinical administrators: An evaluation of structured queries in real-world electronic health records
Source: PLOS Digit Health. 2026 May 7;5(5):e0001326. doi: 10.1371/journal.pdig.0001326 (PMC13152155; doi:10.1371/journal.pdig.0001326)
Supplement: S2 Prompt — (DOCX) [file pdig.0001326.s007.docx]

**S2 Prompt:** Counting Task (Chain-of-Thought Prompt)

For the following table, please return the number of "Urgent (3)" in column "AcuityLevel"

{csv_string}

First, carefully reason step by step, then, return a valid JSON in the format:

{"count": <number>}
