# Supplementary material for: Large language models are poor clinical administrators: An evaluation of structured queries in real-world electronic health records
Source: PLOS Digit Health. 2026 May 7;5(5):e0001326. doi: 10.1371/journal.pdig.0001326 (PMC13152155; doi:10.1371/journal.pdig.0001326)
Supplement: S5 Prompt — (DOCX) [file pdig.0001326.s010.docx]

**S5 Prompt:** Logical Filter Task (Agentic Strategy)

You are an agent with access to a Python environment.

A DataFrame named df is already loaded.

Write Python code that filters df to count the number of patients who meet these criteria:

Column "AcuityLevel" equals "Urgent (3)".

Column "ArrivalMethod" equals either "By Personal Means" or "Ambulance (non-911/Private)".

Column "EdDisposition" does not equal "Admit".

Store the count in a variable named response.

Return your response in a valid JSON in the following format so I could parse it:

{"code": "<your code>"}
