# Supplementary material for: Large language models are poor clinical administrators: An evaluation of structured queries in real-world electronic health records
Source: PLOS Digit Health. 2026 May 7;5(5):e0001326. doi: 10.1371/journal.pdig.0001326 (PMC13152155; doi:10.1371/journal.pdig.0001326)
Supplement: Attachment — Submitted filename: Response to Reviewers.docx [file pdig.0001326.s011.docx]

**Response to Reviewers**

1. Please update your online Competing Interests statement. If you have no competing interests to declare, please state: “The authors have declared that no competing interests exist.”

2. Please provide separate main figure files in .tif or .eps format only and ensure that all files are under our size limit of 10MB. You may leave the embedded figures in the manuscript.

For more information about how to convert your figure files please see our guidelines: https://journals.plos.org/digitalhealth/s/figures

Done

3. We have noticed that you have uploaded Supporting Information files, but you have not included a list of legends. Please add a full list of legends for your Supporting Information files before or after the references list.

Added

4. Some material included in your submission may be copyrighted. According to PLOS’s copyright policy, authors who use figures or other material (e.g., graphics, clipart, maps) from another author or copyright holder must demonstrate or obtain permission to publish this material under the Creative Commons Attribution 4.0 International (CC BY 4.0) License used by PLOS journals. Please closely review the details of PLOS’s copyright requirements here: PLOS Licenses and Copyright. If you need to request permissions from a copyright holder, you may use PLOS's Copyright Content Permission form.

Please respond directly to this email or email the journal office and provide any known details concerning your material's license terms and permissions required for reuse, even if you have not yet obtained copyright permissions or are unsure of your material's copyright compatibility.

Potential Copyright Issues:

Figure 1: Please confirm whether you drew the images / clip-art within the figure panels by hand. If you did not draw the images, please provide (a) a link to the source of the images or icons and their license / terms of use; or (b) written permission from the copyright holder to publish the images or icons under our CC-BY 4.0 license. Alternatively, you may replace the images with open source alternatives. See these open source resources you may use to replace images / clip-art:
- https://commons.wikimedia.org
- <https://openclipart.org/>

Figure 1 was generated using open-source clipart

5. If the reviewer comments include a recommendation to cite specific previously published works, please review and evaluate these publications to determine whether they are relevant and should be cited. There is no requirement to cite these works unless the editor has indicated otherwise.

Noted

**Comments to the Author**

1. Does this manuscript meet PLOS Digital Health’s [publication criteria](https://journals.plos.org/digitalhealth/s/journal-information#loc-criteria-for-publication)? Is the manuscript technically sound, and do the data support the conclusions? The manuscript must describe methodologically and ethically rigorous research with conclusions that are appropriately drawn based on the data presented.

Reviewer #1: Yes

Reviewer #2: Yes

2. Has the statistical analysis been performed appropriately and rigorously?

Reviewer #1: Yes

Reviewer #2: Yes

3. Have the authors made all data underlying the findings in their manuscript fully available (please refer to the Data Availability Statement at the start of the manuscript PDF file)?

The [PLOS Data policy](https://journals.plos.org/digitalhealth/s/data-availability) requires authors to make all data underlying the findings described in their manuscript fully available without restriction, with rare exception. The data should be provided as part of the manuscript or its supporting information, or deposited to a public repository. For example, in addition to summary statistics, the data points behind means, medians and variance measures should be available. If there are restrictions on publicly sharing data—e.g. participant privacy or use of data from a third party—those must be specified.

Reviewer #1: No

Reviewer #2: Yes

4. Is the manuscript presented in an intelligible fashion and written in standard English?

PLOS Digital Health does not copyedit accepted manuscripts, so the language in submitted articles must be clear, correct, and unambiguous. Any typographical or grammatical errors should be corrected at revision, so please note any specific errors here.

Reviewer #1: Yes

Reviewer #2: Yes

5. Review Comments to the Author

Please use the space provided to explain your answers to the questions above. You may also include additional comments for the author, including concerns about dual publication, research ethics, or publication ethics. (Please upload your review as an attachment if it exceeds 20,000 characters)

Reviewer #1: The paper addresses a very interesting and important topic. However, there are a few areas where improvements can be made:
1. Recommendations: Adding a strong takeaway/ recommendation can make an impact. in this case, I suggest adding Model Context Protocol (MCP) as that directly solves the problem being discussed here. For more details check this paper- <https://arxiv.org/html/2507.01053>

Thank you for making this important point. We have emphasized this fact, and the highly-relevant reference, in the conclusion.

“Hybrid frameworks combining LLMs with traditional database or coding strategies, like the Model Context Protocol (MCP)(32), are currently required to achieve practical utility, accuracy, and safety in clinical administration tasks.”

2. The paper can be made more impactful by evaluating the model using standardized criteria developed for LLMs- the TRIPOD-LLM checklist. Here is an easy-to-use official web interface- <https://tripod-llm.vercel.app/>

Thank you for the suggestion. We have filled and added the TRIPOD-LLM checklist to the paper as Supplementary File 1.

“Further study details can be found in the TRIPOD-LLM checklist(23) (**Supplementary File 1**).”

Reviewer #2: Inconsistent Model Naming: The manuscript uses inconsistent names for the Llama models. For example, Table 1 (p. 27) lists "Llama-3.3-8B" and "Llama-3.1-70B," while the text and other tables refer to "Llama-3.1-8B" (p. 11, p. 29) and "Llama-3.3-70B" (p. 12, p. 23). Please audit all model names throughout the manuscript, tables, and figures to ensure they are consistent.

Thank you for pointing out this issue. We have corrected the erroneous entries in the table and ensured consistency of model naming throughout the manuscript and in the figures.

Missing Information in Table 1: The entry for "Distill Qwen-72B" in Table 1 (p. 28) is incomplete. Please provide the missing details for this model.

We have corrected all errors in Table 1.

Clarity of Statistical Tests: The text reports p-values for accuracy comparisons (e.g., "p<0.001" on p. 12), but the specific statistical test used (e.g., z-test for proportions, chi-squared test) is not mentioned. Please specify the statistical test used for these comparisons in the Methods section.

Thank you. Methods now specifies two-sided two-proportion z-tests for accuracy comparisons.

“Between-group differences in accuracy (proportion correct) were tested using two-sided two-proportion z-tests with pooled standard errors (α=0.05).”

Figure 5 X-axis Labels: The x-axis labels in Figure 5 (p. 26) are unreadable. Please regenerate this figure with legible labels corresponding to the models listed in the caption.

We have fixed Figure 5.

Acronym Definition: The acronym "ESI" (Emergency Severity Index) is used in the "Cohort Characteristics" section (p. 11) but is not defined. Please define it at its first use.

We have modified the punctuation in that section to make the definition of ESI clearer:

“Of the ED visits extracted, most patients were triaged as urgent (emergency severity index [ESI] 3, 58.8%), followed by emergent (ESI 2, 19.7%), and less urgent (ESI 4, 17.5%) (Supplemental Table 1).”

Visibility in Figure 3: In Figure 3 (p. 24), the orange marker for "Llama-3.3-70B" is difficult to distinguish, especially in panels C and D. Consider using a more distinct color or marker shape to improve readability.

Thank you for pointing out this issue. Accordingly, we have regenerated Figures 2, 3, and 4 to provide more clearly contrasting colors and different symbols for each variable.

6. PLOS authors have the option to publish the peer review history of their article ([what does this mean?](https://journals.plos.org/digitalhealth/s/editorial-and-peer-review-process#loc-peer-review-history)). If published, this will include your full peer review and any attached files.

Yes


**Do you want your identity to be public for this peer review?** If you choose “no”, your identity will remain anonymous but your review may still be made public.


For information about this choice, including consent withdrawal, please see our [Privacy Policy](https://www.plos.org/privacy-policy).

Reviewer #1: **Yes:** Shrey Lakhotia

Reviewer #2: No
